# Supplementary material for: Myocardial Deformation Analysis in MYBPC3 and MYH7 Related Sarcomeric Hypertrophic Cardiomyopathy—The Graz Hypertrophic Cardiomyopathy Registry
Source: Genes (Basel). 2021 Sep 23;12(10):1469. doi: 10.3390/genes12101469 (PMC8535960; doi:10.3390/genes12101469)
Supplement: Supplementary file 1 [file genes-12-01469-s001.zip › genes-1368024-supplementary.pdf]

Table S1: Truncating pathogenic or likely pathogenic *MYBPC3* (NM_000256.3) mutations.

Nucleotide Variant	Amino Acid Variant	Mutation Type	Sex	N[1]
c.2864_2865del	p.(Pro955ArgfsTer95)	Frameshift	F, M, M	3
c.2864_2865del	p.(Pro955ArgfsTer95)	Frameshift	M	1
c.1574_1580del	p.(Tyr525CysfsTer28)	Frameshift	M*	1
c.3302del	p.(Thr1101LysfsTer88)	Frameshift	M	1
c.2541C>A	p.(Tyr847Ter)	Nonsense	F	1
c.3767_3769del	p.(Thr1256del)	Deletion	M	1
c.927-9G>A	NA	Splice	M	1
c.772G>A	p.(Glu258Lys)	Splice	M	1
c.772G>A	p.(Glu258Lys)	Splice	M	1
c.772G>A	p.(Glu258Lys)	Splice	F	1
c.772G>A	p.(Glu258Lys)	Splice	M, M	2
c.772G>A	p.(Glu258Lys)	Splice	M	1
c.26-2A>G	NA	Splice	F, F, F, F, F	5
c.26-2A>G	NA	Splice	F	1
c.26-2A>G	NA	Splice	M	1
c.26-2A>G	NA	Splice	M	1
c.26-2A>G	NA	Splice	M	1
c.927-10C>A	NA	Splice	M, F	2
c.821+1G>A	NA	Splice	M	1
c.821+1G>A	NA	Splice	F	1
c.821+1G>A	NA	Splice	F	1
c.821+1G>A	NA	Splice	F	1
c.821+1G>A	NA	Splice	M	1
c.1351+2T>C	NA	Splice	M	1
c.505+1G>A	NA	Splice	M	1
c.3190+5G>A	NA	Splice	F	1
c.1227-13G>A	NA	Splice	M	1

[1] Number of affected family members. * Patient with two mutations in *MYBPC3* (see Table A2) Abbreviations: NA, not available; M, male; F, female.

Table S2: Pathogenic or likely pathogenic *MYBPC3* (NM_000256.3) missense mutations.

Nucleotide Variant	Amino Acid Variant	Sex	N[1]
c.1468G>A	p.(Gly490Arg)	M*	1
c.2449C>T	p.(Arg817Trp)	F	1
c.2449C>T	p.(Arg817Trp)	M	1
c.3373G>A	p.(Val1125Met)	F	1
c.1484G>A	p.(Arg495Gln)	M	1

[1] Number of affected family members. * Patient with two mutations in *MYBPC3* (see Table A1). Abbreviations: M, male; F, female.

Table S3: Pathogenic or likely pathogenic *MYH7* (NM_000257.4) mutations.

Nucleotide Variant	Amino Acid Variant	Mutation Type	Sex	N[1]
c.697G>A	p.(Ala233Thr)	Missense	M, F	2
c.697G>A	p.(Ala233Thr)	Missense	F	1
c.1816G>A	p.(Val606Met)	Missense	F, F	2
c.2004C>G	p.(His668Gln)	Missense	M	1
c.1331A>C	p.(Asn444Thr)	Missense	F	1
c.1207C>T	p.(Arg403Trp)	Missense	F	1
c.1063G>A	p.(Ala355Thr)	Missense	F	1
c2606G>A	p.(Arg869His)	Missense	M	1
c.2602G>C	p.(Ala868Pro)	Missense	F	1
c.2218A>G	p.(Lys740Glu)	Missense	F	1
c.2218A>G	p.(Lys740Glu)	Missense	M	1
c.2167C>G	p.(Arg723Gly)	Missense	F	1
c.732+1G>A	p.?	Splicing	M	1
c.2539_2541del	p.(Lys847del)	Small deletion	M	1
NA	NA	NA	F, F	2

[1] Number of affected family members. Abbreviations: NA, not available; M, male; F, female.
